# Noise-precision tradeoff in predicting combinations of mutations and drugs

**DOI:** 10.1371/journal.pcbi.1006956

**Published:** 2019-05-22

**Authors:** Avichai Tendler, Anat Zimmer, Avi Mayo, Uri Alon

**Affiliations:** Dept. Molecular Cell Biology, Weizmann Institute of Science, Rehovot, Israel; Mount Sinai School of Medicine, UNITED STATES

## Abstract

Many biological problems involve the response to multiple perturbations. Examples include response to combinations of many drugs, and the effects of combinations of many mutations. Such problems have an exponentially large space of combinations, which makes it infeasible to cover the entire space experimentally. To overcome this problem, several formulae that predict the effect of drug combinations or fitness landscape values have been proposed. These formulae use the effects of single perturbations and pairs of perturbations to predict triplets and higher order combinations. Interestingly, different formulae perform best on different datasets. Here we use Pareto optimality theory to quantitatively explain why no formula is optimal for all datasets, due to an inherent bias-variance (noise-precision) tradeoff. We calculate the Pareto front of log-linear formulae and find that the optimal formula depends on properties of the dataset: the typical interaction strength and the experimental noise. This study provides an approach to choose a suitable prediction formula for a given dataset, in order to best overcome the combinatorial explosion problem.

## Author summary

Sometimes a combination of drugs works much better than each drug alone. Finding such drug cocktails is a pressing challenge in order to combat drug resistance and to improve drug effects. However, it is impossible to test all combinations of multiple drug experimentally. Therefore, researchers are looking for computational rather than experimental approaches to overcome this problem. One approach is to measure the effect of few drugs and plug it into a formula that predicts the effect of many drugs together. Existing prediction formulae typically perform best on the dataset that they were developed on, but less well on other datasets. Here we explain this observation and give a guide for the choice of an optimal prediction formula for a given dataset. The optimal formula depends on two main properties of the dataset: 1) The interaction strength between the drugs and 2) The experimental noise in the data. This study may help researchers discover effective combinations of multiple drugs and multiple perturbations in general.

## Introduction

Different fields of biology ask how multiple perturbations affect a biological system. For example, to understand the function of DNA sequences such as promoters or coding regions, or to design new ones, it is important to understand how mutations combine to affect function [1–4]. Another widely studied example is how multiple drugs combine to affect cells and organisms. This question is important for developing effective combination therapy [5–9] and to reduce drug resistance [10–14].

A major challenge in these fields is the combinatorial explosion problem: the number of combinations increases exponentially with the number of perturbations. Given *n* different single perturbations, there are 2^*n*^ possible combinations. In DNA sequences there are 4^*n*^ combinations of bases so that sequences of 30bp have 10^18^ possible combination. Drugs present the additional dimension of doses, so that 8 drugs at 6 doses amount to 6^8^≈10^6^ combinations. Therefore, the number of combinations quickly outgrows experimental ability.

To overcome the combinatorial explosion problem, there are two main approaches. In the case of sequences, one can use directed evolution to find sequences with desired function [15–19]. This approach is powerful and is based on exponential expansion of the sequences with highest function. However, experimental evolution still covers only a tiny fraction of sequence space and has the potential to get stuck on local optima. In the case of drugs this approach is not feasible.

The other main approach is to use mathematical models to estimate the effects of combinations using only a small number of measurements. Machine learning studies use regression-like models to estimate drug and mutation effects based on a learning set of measurements [20–24]. For example [4] analyzed combinations of mutations on the lac promoter, and [25] analyzed a library of mutation in green fluorescent protein. One limitation of machine learning is that it requires extensive training data, which may exceed experimental ability when samples are rare and perturbations are costly, as in the case of drug combinations.

To overcome the lack of large training datasets, another line of research establishes analytical formulae to estimate combination effects based on, for example, measurements of single perturbations and pairs. Analytical formulae can include knowledge about the biology of the system and can therefore be more effective than machine learning when data is scarce. The most common baseline model, that seems to work well as a first approximation in many cases, is Bliss independence [26] in which the effect of a pair of perturbations is the product of the single perturbation effects, *s*_*ij*_ = *s*_*i*_*s*_*j*_. Bliss independence is equivalent to additivity in log-effect space. Another baseline model for drugs is Loewe (dose additivity) [27], but seems to be less accurate than the Bliss approximation for high-order drug combinations [28,29].

Baseline models are generally inaccurate because they do not consider the interactions between perturbations. These interactions are called synergy and antagonism, in the case where the combination shows larger or smaller effect than the baseline model, respectively. Several studies have attempted to present formulae that take interactions into account, by including measurements for pairs. Wood et al. [30] introduced an Isserlis-like formula based on singles and pairs. For triplets, the formula is *s*_123_ = *s*_1_*s*_23_+*s*_2_*s*_13_+*s*_3_*s*_12_−2*s*_1_*s*_2_*s*_3_. This formula worked well for combinations of up to 4 antibiotics.

Zimmer et al [31] presented a model which used measurements of dose-response for single drugs and drug pairs to compute the dose-dependent effect of higher order combinations, with excellent accuracy on antibiotics and anti-cancer drugs. An additional formula, based only on pairs, performed well on small single-dose drug datasets [32].

Surveying these studies, it seems that there is no best formula that outperforms others on all datasets. Instead, each formula works well on the dataset it was developed on, but typically less well on other datasets. This situation suggests that, because datasets differ in their noise and interaction strengths, there may be a range of formulae to consider. There is therefore a need to compare formulae, to understand when formulae fail, and to develop ways to decide which formula to use when considering a given dataset.

Here, we address these questions by studying the tradeoffs inherent in formulae for combinations. We study wide classes of formulae and test them on twelve experimental datasets for drugs and sequences, as well as on synthetically generated datasets. We find that no formula outperforms the others on all datasets. Instead, each dataset has a different optimal formula. On the other hand, many formulae are suboptimal for all datasets.

**Fig 1:**
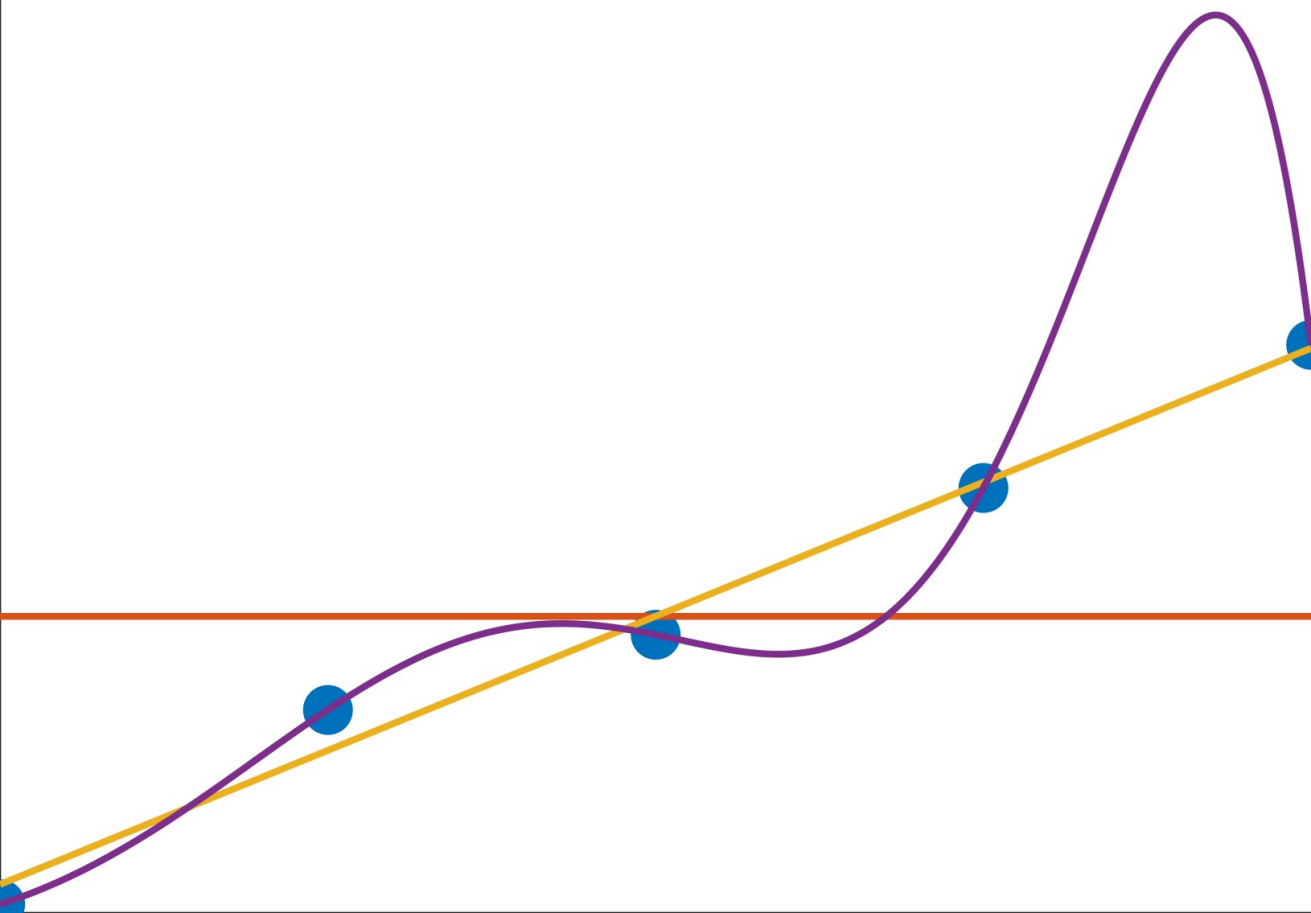
Demonstration of the Bias-Variance tradeoff on a simple example. The data points (blue) originate from a line y = x plus additive noise. Different models were used to fit this data. A low complexity model gives constant prediction, y = a (orange line). Its predictive power is poor because of high bias. A high complexity model fits an 8^th^ order polynomial (purple line) to the data, this model is rich enough to capture precisely all the training points, but its generalization to unseen data is poor due to its high variance. A medium complexity model (yellow line, y = ax+b) gives good predictions for new data points, keeping both bias and variance low.

We explain this result using a well-known concept from statistical learning, the bias-variance tradeoff [33–35]. Roughly speaking, good formulae should be complex or expressive enough to capture the true variability of the dataset (low bias). On the other hand, formulae should be simple enough in order to avoid overfitting the noise in the dataset (Fig 1). Hence, the optimal formula for a dataset should be dependent on the typical effect size (true variability) of the dataset as well as the experimental noise.

In order to understand this tradeoff, we use Pareto optimality [36–39]. Pareto optimality was previously used to study model selection and hyper-parameter choice in machine learning models [40,41]. We use it to define the optimal formula for each dataset, based on its noise and interaction strength. We suggest a method to choose the optimal formula for a new dataset.

## Results

### Family of formulae for predicting triplets from pairs

For simplicity, we concentrate on the problem of predicting the effect of triplets of perturbations from data on the effects of pairs and single perturbations. We provide formula for the effects of k perturbations in the supporting information (S1 Text, [29,42]).

To establish notation and terminology, we use the term perturbation as a general term for drug, mutation or other type of change in the system. We define the effect as the measurable outcome of the perturbations on the system function, such as survival of cancer cells for anti-cancer drugs, growth rate of bacteria in case of antibiotics, and the activity of a promoter or a protein in the case of sequence mutations.

Three different perturbations will be denoted by 1,2,3. The value of the effect in the absence of perturbation (wild-type) is *S*_∅_. The effects of single perturbations are *S*_1_,*S*_2_,*S*_3_, of pairs of perturbation are *S*_12_,*S*_13_,*S*_23_. The effect of the triplet perturbation, which we wish to predict given singles and pairs effect data, is *S*_123_. For the effects normalized by the wild-type we use lower case letters $s_{x}=\frac{S_{x}}{S_{\emptyset }}$

Formulae from the literature include the Bliss independence formula:

$$s_{123}=s_{1}s_{2}s_{3}$$ (1)

Machine learning approaches often use a regression formula:

$$s_{123}=\frac{s_{12}s_{13}s_{23}}{s_{1}s_{2}s_{3}}.$$ (2)

This formula results from regression where one fits the effects of singles and pairs to *s* = ∑_*i*_*a*_*i*_*x*_*i*_+∑_*i*,*j*_*a*_*ij*_*x*_*i*_*x*_*j*_ where *x*_*i*_ = 0 if mutation *i* is absent and *x*_*i*_ = 1 if it is present.

A third formula uses only information from pairs [32]:

$$s_{123}=\sqrt{s_{12}s_{13}s_{23}}.$$ (3)

These formulae belong to the class of log-linear formulae, and hence we focus on this class. The most general formula in this class, taking into account the symmetry in perturbation indices (re-naming drugs 1, 2 and 3 should not affect the prediction for *S*_123_) is:

$$S_{123}=S_{\emptyset }^{\alpha }{\left(S_{1}S_{2}S_{3}\right)}^{\beta }{\left(S_{12}S_{13}S_{23}\right)}^{\gamma }$$

To make the calculation linear, we use the logarithm of the un-normalized effects *L*_*x*_ = log(*S*_*X*_), resulting in

$$L_{123}=\alpha L_{\emptyset }+\beta (L_{1}+L_{2}+L_{3})+\gamma (L_{12}+L_{13}+L_{23})$$

The log-linear formulae thus have three parameters, *α*,*β* and *γ*. They include the previous formula discussed above: Bliss independence is when *α* = −2,*β* = 1,*γ* = 0 and regression is *α* = *γ* = 1,*β* = −1.

### The precision of the log-linear class of models

We now evaluate the precision of each formula. As an operational definition of precision, we use a Taylor-series approach. We assume that the log effect is a smooth function *f* of multiple inner variables of the system. Each perturbation is represented by a change in one of these inner variables.

Without loss of generality, we can assume that without perturbations, *L*_∅_ = *f*(0,0,0). Then *L*_1_, the log effect of perturbation 1, is *L*_1_ = *f*(*x*,0,0), for some value of *x*. Similarly, the other two single perturbations are *L*_2_ = *f*(0,*y*,0) and *L*_3_ = *f*(0,0,*z*). The pair log effects are *L*_12_ = *f*(*x*,*y*,0),*L*_13_ = *f*(*x*,0,*z*),*L*_23_ = *f*(0,*y*,*z*). To predict the triplet, we need to estimate *L*_123_ = *f*(*x*,*y*,*z*). Mathematically, this is equivalent to the question of estimating a function on one vertex of a 3D box given its values on the other 7 vertices [42].

Even though in reality perturbations are sometimes not small, we will next assume that they are in order to give an operationalized and analytically solvable way to discuss precision. When the values of *x*,*y* and *z* are such that they represent small perturbations, one can use a Taylor expansion and ask which of the formulae are precise to which order of expansion (no matter what the exact form of *f*).

Here we will derive conditions for a formula to be precise to the 0^th^, 1^st^ and 2^nd^ orders in Taylor series. But first we explain intuitively what these precisions orders mean. Formulae precise to 0^th^ order have the property that if all effects are equal, *L*_∅_ = *L*_*i*_ = *L*_*ij*_ = *C* the prediction for the triplet is equal to that effect: *L*_123_ = *C*. Formulae accurate to first order have the property that if all pairs are Bliss independent in the sense that *s*_*ij*_ = *s*_*i*_*s*_*j*_, then the predicted triplet is also Bliss independent *s*_123_ = *s*_1_*s*_2_*s*_3_.

We now derive the conditions for precision to different orders. The Taylor expansion of *L*_123_ is, to first order:

$$L_{123}=f(x,y,z)=f(0,0,0)+\frac{\partial f}{\partial x}(0,0,0)x+\frac{\partial f}{\partial y}(0,0,0)y+\frac{\partial f}{\partial z}(0,0,0)z+o(|x|,|y|,|z|)$$

We equate this to the Taylor expansion of the log-linear formula:

$$\begin{array}{c} \alpha L_{\emptyset }+\beta (L_{1}+L_{2}+L_{3})+\gamma (L_{12}+L_{13}+L_{23})=\\ =\alpha f(0,0,0)+\beta [f(x,0,0)+f(0,y,0)+f(0,0,z)]+\gamma [f(x,y,0)+f(x,0,z)+f(0,y,z)]=\\ =(\alpha +3\beta +3\gamma )f(0,0,0)+(\beta +2\gamma )\left[\frac{\partial f}{\partial x}(0,0,0)x+\frac{\partial f}{\partial y}(0,0,0)y+\frac{\partial f}{\partial z}(0,0,0)z\right]+o(|x|,|y|,|z|) \end{array}$$

We therefore obtain the condition for a formula to be precise to 0^th^ order:

1=α+3β+3γ

From now on, we restrict ourselves to the class of models that are precise to 0^th^ order. We next ask which models are precise to 1^st^ order. The condition for 1^st^ order precision is:

β+2γ=1

All the formulae on this line in beta-gamma space give exact approximation to the first order. For example, the Bliss (*β* = 1,*γ* = 0), the regression (*β* = −1,*γ* = 1) and pairs formula $\left(\beta =0,\gamma =\frac{1}{2}\right)$ fall on this line of first order precision.

We can define the deviation from 1^st^ order precision as follows:

$$P_{1st}(\alpha ,\beta ,\gamma )={\left(1-\beta -2\gamma \right)}^{2}$$

We next ask which formulae are precise to the second order. We find that there is only one log-linear formula which is precise to the second order–the regression formula of Eq 2 (S1 Text) (*α* = *γ* = 1,*β* = −1). The deviation of other formula from second-order precision can be represented by the sum of the coefficients of the second order error (S1 Text):

$$P_{2nd}(\alpha ,\beta ,\gamma )={\left(\frac{1}{2}-\frac{\beta }{2}-\gamma \right)}^{2}+{\left(1-\gamma \right)}^{2}$$

**Fig 2:**
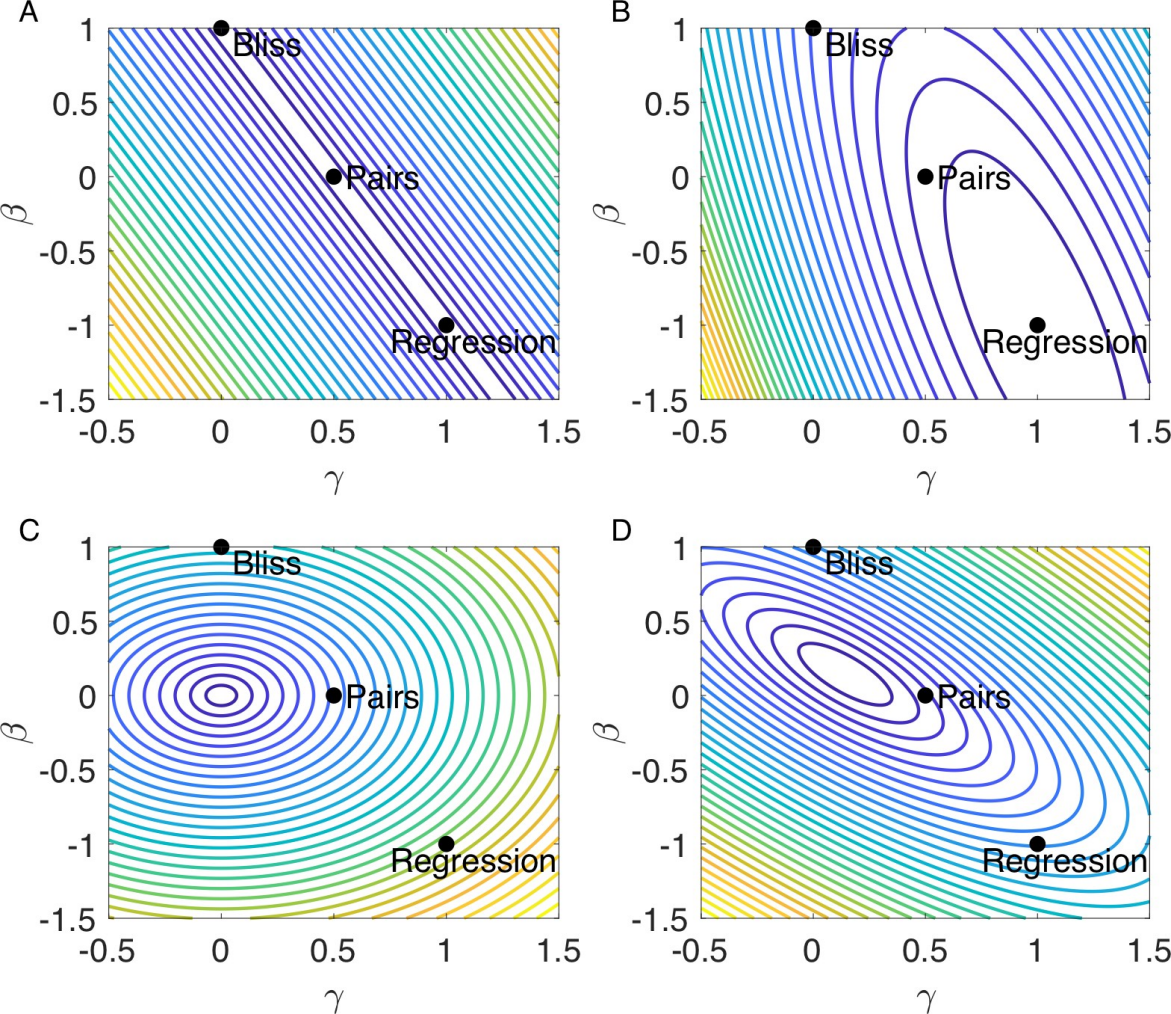
Performance functions in log-linear model space for the tasks of noise and precision. **(A)** First-order-precision performance function contours show a line of formulae which are accurate to the first order, including Bliss, pairs, regression and other formulae. **(B)** Second order precision performance function contours show that only formula accurate to the second order is the regression formula. **(C)** Noise performance function contours in case there is no noise in the wild type measurement, the noise is zero at *β*= 0,*γ* = 0. **(D)** Noise performance function contours in case there is noise in the wild type measurement, the noise is minimal at $\alpha =\beta =\gamma =\frac{1}{7}$.

The precision findings are summarized in (Fig 2A and 2B). The figures plot contours of accuracy to different orders as a function of *β* and *γ*. In the plots, *α* is evaluated by the zero-order precision demand *α* = 1−3*β*−3*γ*. The plots are therefore restricted to 0^th^ order precise formulae. It is seen that optimal first-order accuracy occurs on a line in model space which includes the Bliss and regression models, and that second-order precision has elliptical contours maximal at the regression model.

### Formulae differ in their robustness to experimental noise

If precision was the only factor at play, one would expect the regression model to outperform others. However in most real datasets this model does poorly [31]. The reason is that it is sensitive to experimental noise. To estimate the robustness to noise of different models, we model experimental noise in the measured effects, *L*_*i*_ = *L*_*i*_+*χ*_*i*_ and *L*_*ij*_ = *L*_*ij*_+*χ*_*ij*_, where χ are independent Gaussian noise with equal STD σ for all measurements (similar conclusions apply to the case of non-independent noise, S1 Text). This corresponds to log-normal multiplicative noise for the effect measurements. Such log-normal noise is typical for experiments on drug and mutation effects [31,32].

Here we derive an expression for the noise in the predicted triplet effect. We must separate between two cases. Case I occurs when there is experimental noise in *L*_∅_ (the wild-type), as is the typical case for sequence (mutation) data, so that *L*_∅_ = *L*_∅_+*χ*_∅_. Case II is when *L*_∅_ is noiseless, as often happens for drug combinations when the effect is cell survival and *L*_∅_ = 0 by definition.

To compute the variation in the prediction of a triplet *s*_123_ given the noise in the pair and single inputs, we assume independent noise for each variable. The noise (std) for case I depends on the three parameters of the model *α*,*β* and *γ*:

$$P_{N,WTnoise}(\alpha ,\beta ,\gamma )=\sigma \sqrt{\alpha^{2}+3\beta^{2}+3\gamma^{2}}$$

And in case II (noiseless *L*_∅_) only on the parameters *β* and *γ*:

$$P_{N,WT=1}(\alpha ,\beta ,\gamma )=\sigma \sqrt{3\beta^{2}+3\gamma^{2}}$$

Note that noise is minimal when *α* = *β* = *γ* = 0, a formula that always predicts 0. This model is not precise even to 0^th^ order. Considering only models precise to 0^th^ order, we obtain the minima of the noise performance function in case of noisy wild type (S1 Text):

$${argmin(P}_{N,WTnoise}(\alpha ,\beta ,\gamma ))=(\frac{1}{7},\frac{1}{7},\frac{1}{7})$$

Which simply averages the inputs *L*_∅_,*L*_*i*_ and *L*_*ij*_, and in case of noiseless wild-type simply taking the wild-type value *S*_∅_ as the prediction

$${argmin(P}_{N,WT=1}(\alpha ,\beta ,\gamma ))=(1,0,0)$$

Contours of this function in the cases of presence and absence of noise in the wild-type appear in (Fig 2C and 2D). In both cases noise grows with distance from the single minimum.

### Computing the noise-1^st^ order–precision Pareto front

In order to compare models according to the two tasks, precision and noise, we use the Pareto front approach. The Pareto front is defined as the set of formula for which there is no other formula that is better at both tasks. Given the two performance functions of noise and precision, we compute the Pareto front as the set of points of external tangency of the performance contours [43,44]. The resulting front is a one-dimensional curve in the space of formulae (beta-gamma space). In the case of first-order precision and noise robustness, the front is a straight line.

**Fig 3:**
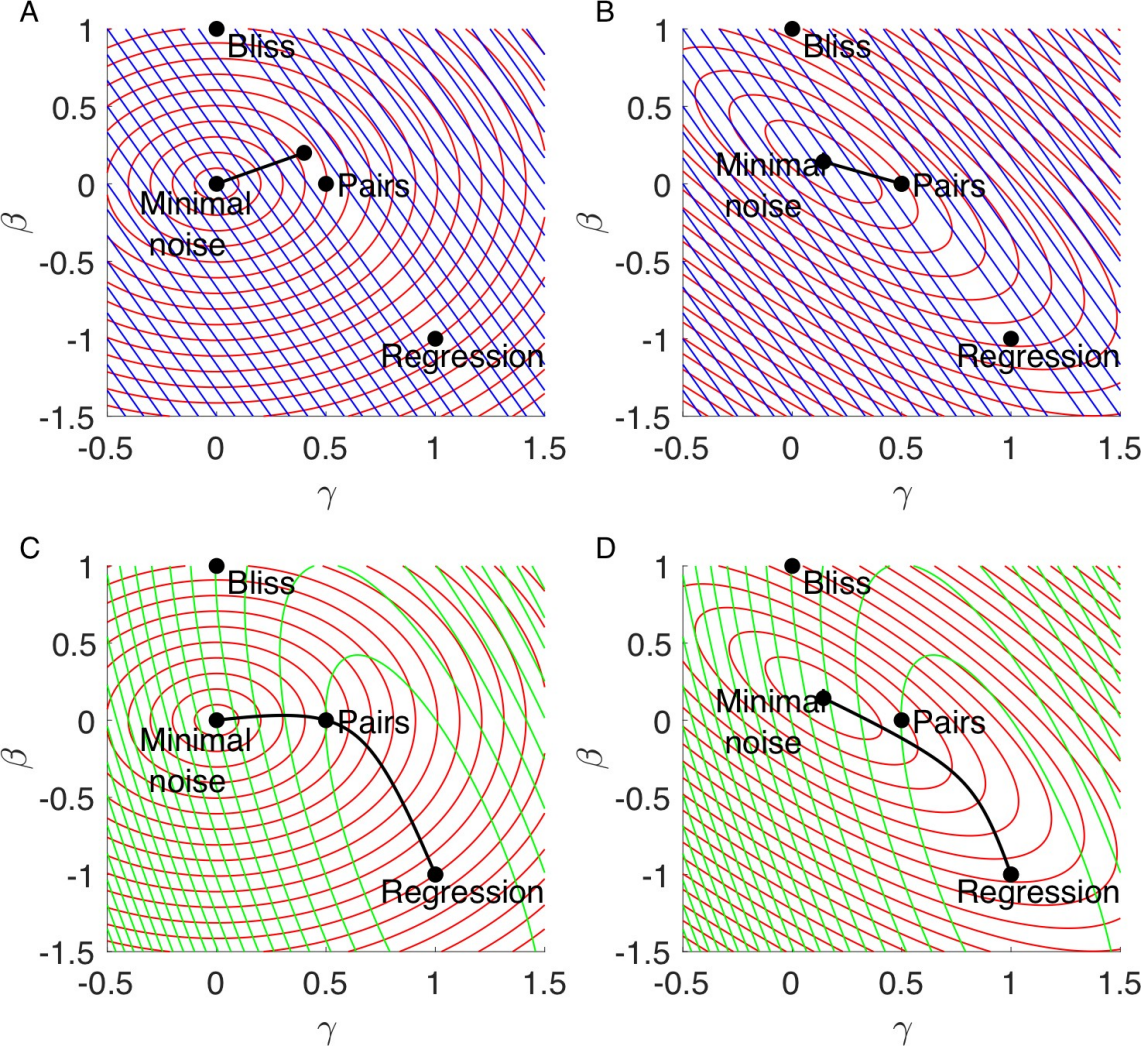
Pareto fronts of pair of performance functions fall on lines. In all subfigures red lines are contours of noise performance function, blue lines are contours of first order accuracy performance function, green lines are contours of second order accuracy performance function and the a black line is the Pareto front. The Pareto fronts are computed as the set of points where the contours of the two performance functions are parallel. **(A)** Contours of noise and first order accuracy when there is no noise in wild-type measurement, the Pareto front is the line between the minimal noise formula in the LHS and the minimal noise formula among first order accurate formula in the RHS. **(B)** Same as (A) in case there is noise in wild-type measurement, in this case the minimal noise formula accurate to the first order is the pairs formula. **(C)** Contours of noise and second order accuracy when there is no noise in wild-type measurement, the Pareto front is a curve between the minimal noise formula in the LHS and the regression formula, which is exact to the second order in the RHS. **(D)** Same as (C) in case there is noise in wild-type measurement.

In the absence of wild-type noise the Pareto front is defined by (see S1 Text and Fig 3A):

γ=2β

Or in the presence of wild-type noise (see S1 Text and Fig 3B):

5β+2γ=1

If noise and first-order precision are the only tasks faced by formulae, it is expected that all optimal formulae will fall on this line.

### Computing the noise-2^nd^ order Pareto front

We next computed the Pareto front where the two tasks are noise robustness and second order precision. In the case of noiseless wild-type, this give the conic defined by the equation (see S1 Text and Fig 3C):

$$-2\gamma^{2}+7\beta \gamma +2\beta^{2}+\gamma -6\beta =0$$

In the case of noisy wild-type we find (see S1 Text and Fig 3D):

$$5\beta^{2}+28\beta \gamma -22\beta +16\gamma^{2}-20\gamma +5=0$$

### Computing the entire Pareto front

**Fig 4:**
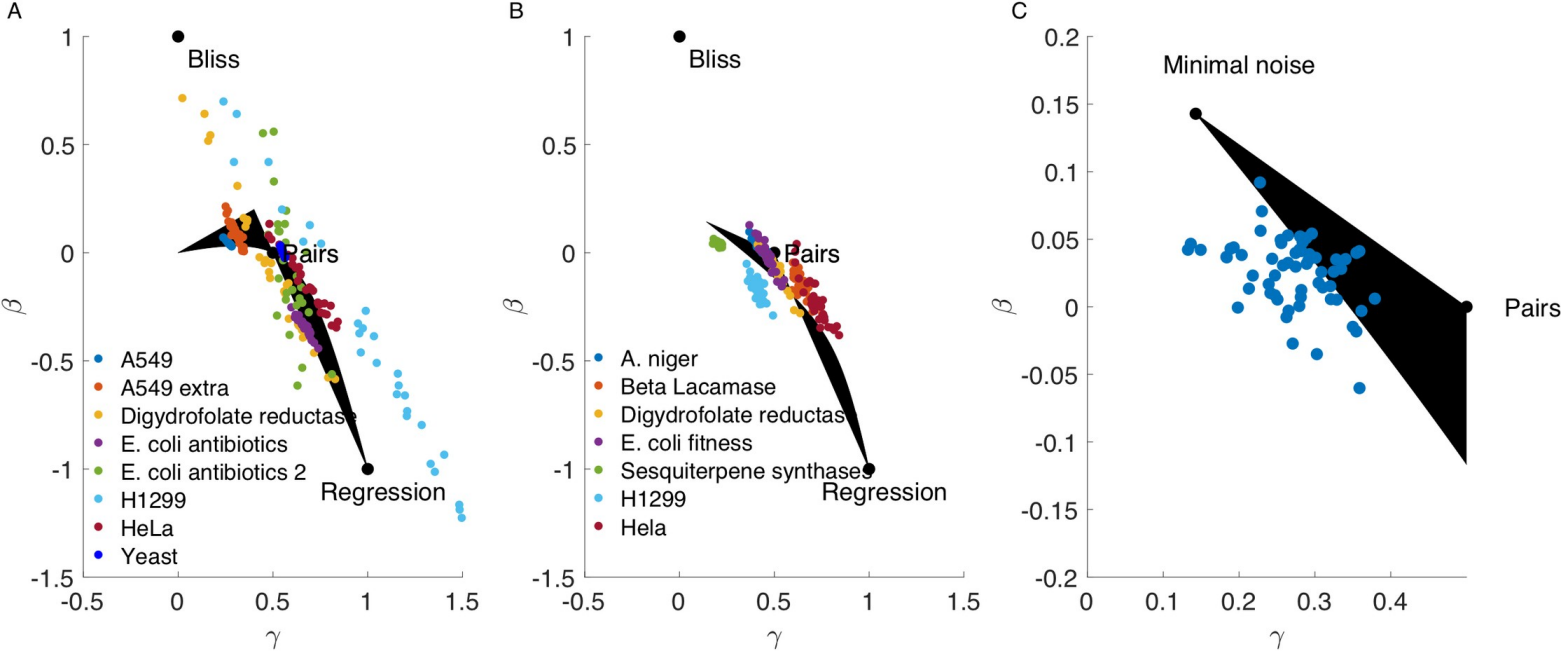
Best formulae for real datasets fall near the Pareto front for the three tasks. The Pareto front is the black area, each point is the optimal formula (in terms of RMSE) for a given dataset. Points of the same color correspond to different bootstrapped samplings of the same dataset. Datasets are detailed in Table 1. Subplots correspond to the two cases: **(A)** there is no noise in the wild-type measurement *S*_∅_. **(B)** noise exists in *S*_∅_ (includes mutation datasets and drug datasets in which we used the expansion trick). **(C)** UniProbe datasets fall on the Pareto front close to the noise archetype. Each blue point corresponds to the best formula for a different dataset on the effect of mutations on protein binding from the UniProbe database. For each dataset we used 1000 randomly selected wild-types and triplet mutations on those wild-type backgrounds to generate the dataset on which we checked the formulae. Points fall close to the noise robustness archetype.

It is now possible to compute the entire Pareto front which consists of optimizing the three performances together. The boundary of the Pareto front is defined by the Pareto fronts of the pairs of tasks. The entire Pareto front in the cases of noiseless and noisy wild-type is composed of two thin triangle-like shapes that meet at a vertex, as shown in Fig 4A and 4B (black region).

We note in passing that the typical solution for a Pareto front with three tasks resembles a single triangle with the optima for the three tasks at the three vertices[43,44]; the elongated two-shape pattern found here results from the fact that the optima for one task, first order precision, falls on a line and not a single point.

The present approach can be applied to any class of formulae. To illustrate this we compute the Pareto front for a class of generalized mean formulae in S1 Text.

### For real datasets the optimal formulae are on the Pareto front

In order to test the relevance of the Pareto front to real data, we compiled a set of thirteen published experimental datasets for drugs and mutations (Table 1). This includes data on the effects of drugs (antibiotics, cancer drugs) on cells, and the effect of mutations on proteins and organisms. The datasets include the effects of singles, pairs and triplets of perturbations. For each dataset, we scanned formulae (scanning *β* and *γ* with *α* = 1−3*β*−3*γ* to provide 0^th^ order precision) and found the formula that gives the smallest root-mean-square error for triplet predictions. This formula, a point in the *β*,*γ* plane, is the optimal formula for that dataset. In order to control for outliers and variation in the data, we repeated this for each dataset on 30 bootstrapped datasets, in which we built a new dataset sampled from the original data with replacements. Thus, each dataset yields 30 additional optimal formula points.

**Table 1:** Datasets used in this paper.

System Name	Perturbation	Effect	Number of data points [expanded]	Details about the data	Reference
A549 (cancer cell line)	Drugs	Cell Survival	896	Combinations of 3 anti cancer drugs in 8 doses.	[50]
A549 extra (cancer cell line)	Drugs	Cell Survival	192		[50] Doses not included in the paper
Aspergilus niger (Fungus)	Mutations	Growth rate	[486]	Mutations in 8 locations.	[51]
Beta lactamase (Bacterium)	Mutations in active site	Cefotaxine resistance	[40]	Fully factorial dataset of mutations in 5 locations.	[52]
Digydrofolate reductase (Protozoan)	Mutations in active site	Pyrimethamine resistance	10 [40]	4 amino acid replacements.	[53]
E. coli antibiotics (bacterium)	Antibiotics	Exponential growth rate	1232	8 drugs at various dosages.	[30]
E. coli antibiotics 2 (bacterium)	Antibiotics	Exponential growth rate	20	6 drugs.	[54]
E. coli fitness (bacterium)	Mutations	Growth rate	[40]	Fully factorial dataset of mutations in 5 locations.	[55]
HeLa (cancer cell line)	Drugs	Cell Survival	16 [52]	Fully factorial dataset of 6 drugs.	[32]
H1299 (cancer cell line)	Drugs	Cell Survival	20 [160]	Fully factorial dataset of 6 drugs.	[32]
Sesquiterpene synthases (enzyme)	Mutations	Catalysis of substartes	[1793]	Fully factorial dataset on 9 sites.	[56]
UniProbe (DNA sequence)	Mutations	Protein binding	61 datasets, 1000 data points randomly sampled from each	Each dataset is fully factorial on 8 sites.	[46]
Yeast	Gene deletions	Growth rate	142442	Triplets were selected based on a former pairs experiment	[49,57]

This is a short summary of properties of the datasets used in this paper. In the number of triplet column numbers in brackets refer to expanded form of a dataset, a form in which different measurements were used as wild-types, in the expanded form we have *S*_∅_≠1.

We find that the optimal formula for all datasets lie close to the Pareto front (Fig 4A). The large datasets fall neatly on the Pareto front (*E*. *coli* antibiotics 1, A549 and others), whereas smaller datasets tend to deviate more due to their larger bootstrapping variance (Dihydrofolate reductase, H1299, *E*. *coli* antibiotics 2). Note also that the main direction of variability of the bootstrapping distribution is parallel to the Pareto front [45].

**Fig 5:**
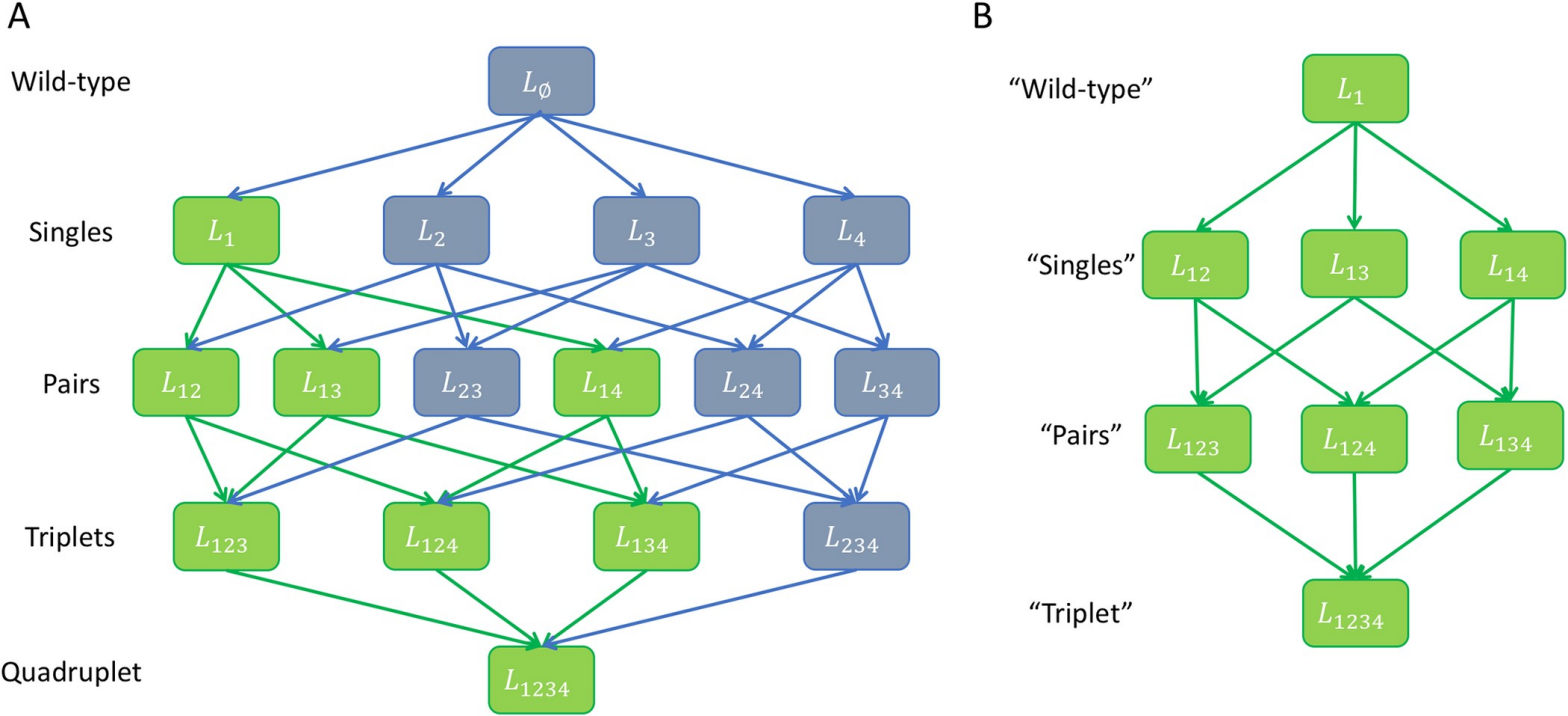
Example of the expansion method to obtain three-pertrubation inetractions from small combinarorial datasets. **(A)** A graph of a fully factorial dataset on four perturbations. In the exansion method, one uses a perturbed system as the wildtype. For example, an expanded triplet is marked in green. It uses *L*_1_ as the wild-type and uses additional perturbations on this background. **(B)** Thus original pairs are now singles, original triplets are now pairs, and the original quadruplet is a triplet.

In the presence of noise in the measured wild-type effect (case II above), the datasets also fall on the Pareto front (Fig 4B). In this case the datasets are larger, hence they have less variability in the bootstrapping. Here, we used an expansion trick to increase the amount of usable data from small fully-factorial datasets. In the expansion trick, we consider treatment with a single perturbation *L*_*i*_ as wild-type *L*_∅_. We then consider treatments with an additional second perturbation *L*_*ij*_ as a single perturbation on the wild-type background, *L*_*i*_, treatments with three perturbations *L*_*ijk*_ as the pairs *L*_*jk*_, in order to predict the triplet *L*_*jkm*_ given by the quadruplet *L*_*ijkm*_ in the original data (Fig 5). We also used pairs and higher order combinations as wildtype to the extent allowed by the dataset. This increases the number of triplets in the fully factorial dataset of order *k* from (k2) to at most (k2)2k in its most extended form (Table 1 shows both original and expanded triplet number).

We further tested 61 datasets from the UniProbe database [46] on protein-DNA binding interactions in fully factorial datasets of 8 mutations. We use the expansion trick using 1000 randomly chosen starting point sequences as a wild-type from each fully factorial dataset. We find that the optimal formulae for these datasets all fall close to the Pareto front (Fig 4C). The results are near the noise-robustness archetype, suggesting that noise is a dominant source of variation in these protein-binding microarray experiments.

### A choice of a formula based on properties of the dataset

We see that optimal formulae for different datasets are close to the Pareto front. We next asked how the properties of the dataset affect which formula is optimal for that dataset. To do so, we generated synthetic datasets with different parameters, so that we could control the level of noise and the level of interaction strength (deviation from the Bliss formula, see S1 Text), the two factors that influence the performance of the formula.

To generate simulated data we used a third order polynomial *f*(*x*,*y*,*z*) with random coefficients, sampled at different random points, with Gaussian noise added (which varies between datasets). The goal is to predict triplets from pairs and singles, that is to predict *f*(*x*,*y*,*z*) from the projections on axes and planes (S1 Text) e.g. *f*(*x*,0,0), *f*(*x*,*y*,0) etc. The noise amplitude of each dataset is the standard deviation of the Gaussian noise added to log effect. The interaction strength (that is synergy/antagonism) of each dataset is given by its mean deviation from the Bliss approximation $I=\frac{\left|s_{ij}-s_{i}s_{j}\right|}{\left|s_{i}s_{j}\right|}$. To control I, we sampled the function at various distance from the origin (S1 Text), where the larger x y and z, the larger the nonlinearity and hence the interaction.

**Fig 6:**
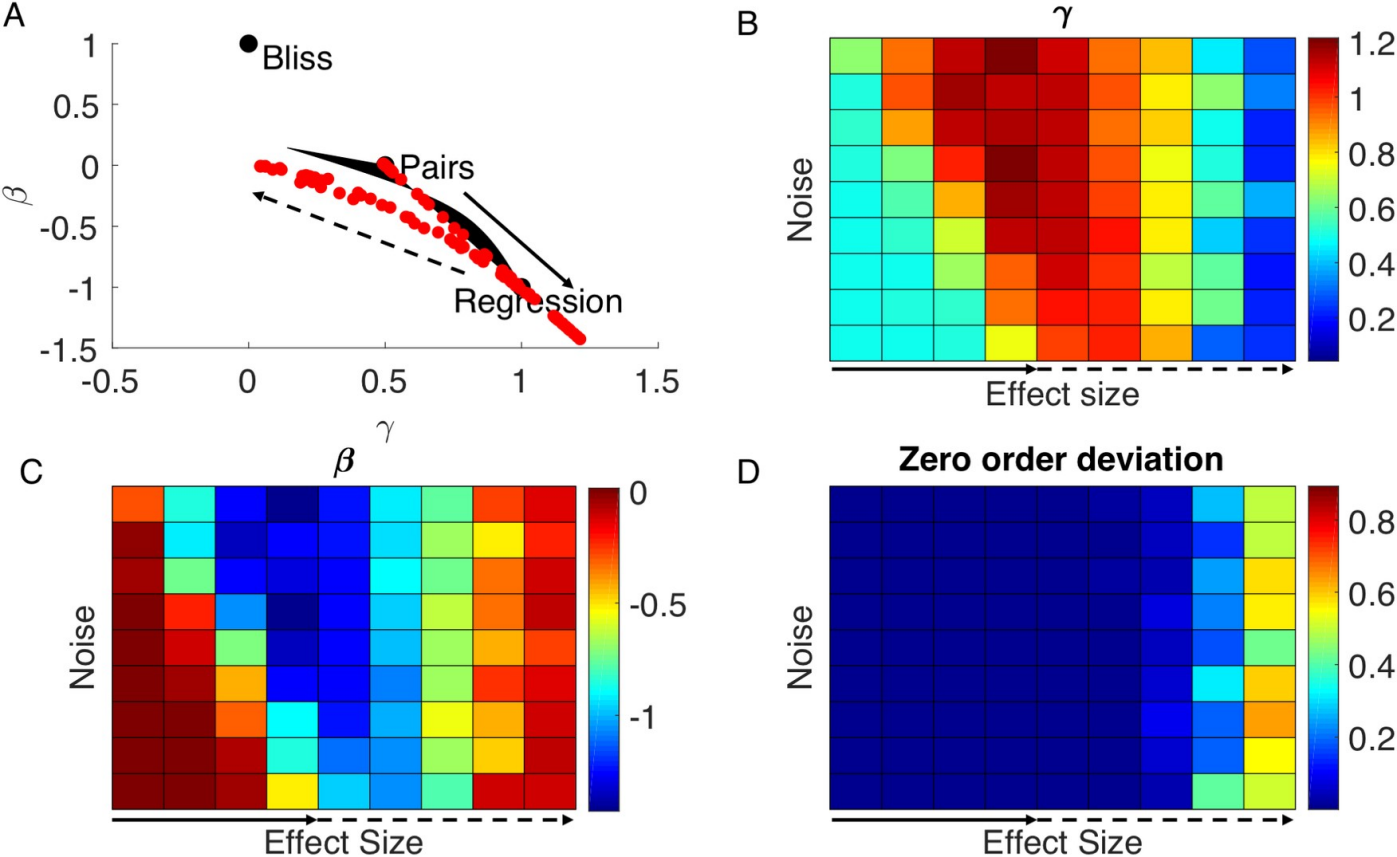
Simulated data fall close to the Pareto front. We generated simulated datasets using random polynomial functions of three variables. Datasets vary by their noise and interaction strength. **(A)** Optimal formula for the simulated datasets fall close to the Pareto front. When interaction strength is increased and noise decrease, points tend towards the second order precision archetype (solid arrow). When interaction strength increases further, points go back towards the noise direction (dashed arrow). **(B)** The value of *γ* for the optimal formula of simulated datasets as function of their noise and interaction strength parameters. Above the solid arrow (which corresponds to the solid arrow of (A)), *γ* increases with interaction strength and decreases with noise, as expected from the noise-precision tradeoff we suggested. Above the dashed arrow (which corresponds to the dashed arrow of (A)), *γ* decreases with interaction strength. The reason is deviation from zero order accuracy which we assume here. **(C)** same as (B) but for the parameter *β*. Here also, results above solid arrow is expected from the trade-off, result above dashed line is explained by deviation from zero order accuracy. **(D)** Deviation from zero order accuracy. Our theory assumes that the optimal formula should be accurate to zero order. We see that in the small interaction strength regime this is indeed the case, while in the large interaction strength (dashed arrow), the optimal regression formula is no longer accurate to order zero.

For each such synthetic dataset, we computed its optimal formula among the log-linear family and found that for datasets with small interaction strengths, the optimal formula falls close to the curve defining the Pareto front (Fig 6A). Interestingly, when interaction strength become larger, points go a bit beyond the second order precision archetype (Fig 6A, solid arrow), and when interaction strength was increased even further, points start to go back to the (0,0) point deviating from the Pareto front (Fig 6A, dashed arrow).

To see the trends described above we plotted the optimal values of *γ* and *β* as function of noise and interaction strength (Fig 6B and 6C). We start by considering the region above the solid arrow (small interaction strength), we see that in this region *γ* increases and *β* decreases with interaction strength. This is the expected result since larger *γ* and smaller *β* means getting closer to the second-order-precision archetype. Second-order precision becomes more important relative to noise as interaction strength gets larger, noise robustness becomes more important than second order precision when the noise in dataset is larger. These results summarize the prediction of Pareto optimality theory.

Interestingly, there are simulated datasets for which the optimal formulae are beyond the second order archetype (Fig 6A, right side). It was found that formulae of second order tend to approximate higher order interactions better than expected [47]. The points beyond the Pareto front are example of formulae of second order which are especially better in predicting the value of the third order function.

In the region of large interaction strength (Fig 6B and 6C over the dashed arrow and, Fig 6A dashed arrow), we see the opposite trend of decreasing *γ* and increasing *β* with interaction strength. The explanation of this surprising result is that formulae in this region no longer satisfy the assumption of precision to 0^th^ order. The interaction strengths in this case are so large, such that the Taylor approximation approach no longer gives the optimal formulae. Fig 6D shows that indeed the formulae found for higher interaction strength no longer gives predictions which are accurate to the 0^th^ order.

These results indicate that one can predict the optimal formula for a dataset if one can estimate its noise and interaction strengths.

## Estimating triplet interactions requires an accurate null model

One general question is to what extent high order interactions exist in biological systems that can’t be explained by pairs. High order interactions in this context are defined as the deviation of the measurement form a null model that includes the effects of single and pair perturbations. Thus, choice of null model can affect the results.

For example, a standard definition of pairwise interaction is:

$$\epsilon_{12}=s_{12}-s_{1}s_{2}$$

This formula is based on a Bliss independence null model for the combined single effects: *s*_12_ = *s*_1_*s*_2_.

Different studies of triplets use different null models [48,49]. For example, a recently study measured the effects of about 150,000 triple gene deletions in yeast, and compared them to single and pair deletions [49]. Third-order interactions were estimated using an Isserlis null model (*s*_123_ = *s*_1_*s*_23_+*s*_2_*s*_13_+*s*_3_*s*_12_−2*s*_1_*s*_2_*s*_3_) yielding

$$\epsilon_{123}={s_{123}-s}_{1}s_{2}s_{3}-\epsilon_{12}s_{3}-\epsilon_{13}s_{2}-\epsilon_{23}s_{1}$$

Evaluating the triplet interaction using the absolute value of *ϵ*_123_ as defined above gives a mean absolute triplet interaction of 0.044. Significant triplet interactions were estimated to be about 100 times more common than significant pair interactions.

We used the present approach to predict the optimal model using the noise and effect size in the pair measurements in this study. The best null model is similar to the pairs model (Eq 3), which is less noise-prone than the Isserlis model. With this null model, the mean absolute triplet effect is 23% lower.

## Discussion

In this study, we find that the problem of predicting the combined effects of perturbations does not have a unique optimal solution. Instead, different solutions and formulae are optimal for different datasets. We analyze the Pareto front of models that trade-off noise robustness and precision. This Pareto front of optimal formulae matches observations on the best formula for a range of real and synthetic datasets.

The present study offers a way to predict the best formula based on the noise and effect size of pairs data. By measuring interaction strength based on pairs, and experimental noise using repeats, one can judge where on the Pareto front the optimal formula might lie for a given dataset.

One important use of these formula is to estimate high-order effects between genes. For example, a third-order effect *ϵ*_123_ is defined by the measured effect of three perturbations minus a null model based on single and pair perturbations. The better the null model, the more accurate the estimation of the high-order effect. We find that the present approach can improve the null model used for estimating the effects of triple yeast gene deletions in a recent large scale study (Kuzmin,2018). The improved estimation lowers the number of apparent three-gene interactions that can’t be explained by pairs. This is relevant for the design of systematic gene perturbation experiments, because it indicates that pairs may be enough to capture most of the interactions. Pair scans are much more feasible than triple-perturbation scans, suggesting an optimistic outlook for understanding complex gene interactions.

This study used a Taylor expansion to define precision. Taylor series strictly apply only to small perturbations. Despite this limitation the method seems to work well for mutation and drug combination dataset. One reason for this is that higher-order effects in biological systems seem to be smaller than low order ones [29], which is equivalent to the underlying assumption of the Taylor approximation. It would be fascinating to use the present approach to analyze additional classes of formula, and to understand the effects of multiple perturbations on additional biological systems.

## Methods

Computations of the maxima of the different performance functions (Fig 2), and the Pareto fronts of multiple performance functions (Figs 3 and 4) were performed analytically, and are detailed in the result section and S1 Text.

All simulations and Figs 1–4 and 6 were produced using MATLAB 2017.

Evaluation metric for formulae performance was RMSE. Therefore, the coefficients of the optimal formula were computed using linear regression on a dataset (Figs 4 and 6).

In Fig 6, simulated data was generated using random symmetric polynomials of degree 3 according to the formula:

$$f(x,y,z)=a_{0}+a_{1}(x+y+z)+a_{2}(x^{2}+y^{2}+z^{2})+a_{3}(x^{3}+y^{3}+z^{3})+a_{4}xyz+a_{5}(xy+xz+yz)+a_{6}(x^{2}y+y^{2}x+x^{2}z+z^{2}x+y^{2}z+z^{2}y)$$

Where *a*_*i*_ were sampled randomly and uniformly between 0 and 1. To get the simulated dataset such random formulae were evaluated at random points in the box [0,*ϵ*]×[0,*ϵ*]×[0,*ϵ*]. The approximation distance *ϵ* varied logarithmically between [0.06,0.06∙2^9^]. To the synthetic dataset we added random log-normal noise *N*(0,*σ*), where *σ* varied logarithmically between [0.01,0.01∙2^9^]. Each point in Fig 6 is based on average of 10 different simulated dataset generated from 10 different random functions, each simulated dataset consists of 300 points.

## Supporting information

S1 Text Supporting information for noise-precision tradeoff in predicting combinations of mutations and drugs. A file with all supporting computations and figures for this paper.
(PDF)

S1 Data Datasets. A zip file containing all datasets used in this paper.
(ZIP)
