# Supplementary material for: Noise-precision tradeoff in predicting combinations of mutations and drugs
Source: PLoS Comput Biol. 2019 May 22;15(5):e1006956. doi: 10.1371/journal.pcbi.1006956 (PMC6548401; doi:10.1371/journal.pcbi.1006956)
Supplement: S1 Text — A file with all supporting computations and figures for this paper. (PDF) [file pcbi.1006956.s001.pdf]

S1 Text. Supporting information for noise-precision tradeoff in predicting combinations of mutations and drugs

Table of Contents

Supporting information for noise-precision tradeoff in predicting combinations of mutations and drugs ***1***

S1. The drug combination problem as approximating a function **2**

Approximating to zeroth order 2

Approximating to first order 2

Approximating to second order 2

S2. Minimum of noise performance function **3**

S3. The Pareto front of noise and first order precision **4**

S4. The Pareto front of noise and second order precision **5**

S5. Performance function for non-independent measurement noise **6**

S6. Generalization to combinations of higher order **6**

S7. Synthetically generated datasets **7**

S8. The bias-variance tradeoff in the generalized mean class of formulae **8**

References **10**

S1. The drug combination problem as approximating a function

We model problem of drug combination as predicting the value of a function $f(x, y, z)$ given the values of $f(0,0,0)$- no drug. $f(x, 0,0), f(0, y, 0), f(0,0, z)$- singles and $f(x, y, 0), f(x, 0, z), f(0, y, z)$- pair measurements.

The log linear formulae therefore correspond to the following approximation:

$$f(x, y, z) \approx$$

$$\alpha f(0,0,0) + \beta\big(f(x, 0,0) + f(0, y, 0) + f(0,0, z)\big) + \gamma(f(x, y, 0) + f(x, 0, z) + f(0, y, z))$$

Approximating to zeroth order

Now we want to find the condition that this equality is true to zeroth order, we take Taylor expansions to zeroth order of both sides to get:

$$f(0,0,0) = \alpha f(0,0,0) + 3\beta f(0,0,0) + 3\gamma f(0,0,0)$$

And the condition for a formula to be precise to zeroth order is:

$$1 = \alpha + 3\beta + 3\gamma$$

This is how we added the $S_\emptyset$ terms to the standard formula – we require that the formula will satisfy the above equation so it will be precise to the zeroth order.

Approximating to first order

We can take the first order approximation (ignoring zeroth order), in the left side $f(x, y, z)$ we get:

$$\frac{\partial f}{\partial x}(0,0,0)x + \frac{\partial f}{\partial y}(0,0,0)y + \frac{\partial f}{\partial z}(0,0,0)z$$

Taking the first order Taylor approximation of the right side:

$$\beta\left(\frac{\partial f}{\partial x}(0,0,0)x + \frac{\partial f}{\partial y}(0,0,0)y + \frac{\partial f}{\partial z}(0,0,0)\right) + 2\gamma\left(\frac{\partial f}{\partial x}(0,0,0)x + \frac{\partial f}{\partial y}(0,0,0)y + \frac{\partial f}{\partial z}(0,0,0)\right)$$

Therefore the deviation of a log-linear formula in the first order Taylor is proportional to:

$$P_{1st}(\alpha, \beta, \gamma) = |1 - \beta - 2\gamma|$$

Approximating to second order

We can continue to approximate a function to the second order, the left side will have the form (only second order, all derivative are taken at (0,0,0)):

$$\frac{1}{2}\left(\frac{\partial^2 f}{\partial x^2}x^2 + \frac{\partial^2 f}{\partial y^2}y^2 + \frac{\partial^2 f}{\partial z^2}z^2\right) + \left(\frac{\partial f}{\partial x}\frac{\partial f}{\partial y}xy + \frac{\partial f}{\partial x}\frac{\partial f}{\partial z}xz + \frac{\partial f}{\partial y}\frac{\partial f}{\partial z}yz\right)$$

For a log linear formula on the right side we get:

$$\frac{\beta}{2}\left(\frac{\partial^2 f}{\partial x^2}x^2+\frac{\partial^2 f}{\partial y^2}y^2+\frac{\partial^2 f}{\partial z^2}z^2\right)+$$

$$+\gamma\left(\frac{\partial^2 f}{\partial x^2}x^2+\frac{\partial^2 f}{\partial y^2}y^2+\frac{\partial^2 f}{\partial z^2}z^2\right)+\gamma\left(\frac{\partial f}{\partial x}\frac{\partial f}{\partial y}xy+\frac{\partial f}{\partial x}\frac{\partial f}{\partial z}xz+\frac{\partial f}{\partial y}\frac{\partial f}{\partial z}yz\right)$$

If we treat second derivatives and mixed derivative as more or less of the same size, we get that the deviation is proportional to:

$$P_{2nd,alternative}(\alpha,\beta,\gamma)=|\frac{1}{2}-\frac{\beta}{2}-\gamma|+|1-\gamma|$$

Or since the terms adds incoherently, it makes sense to take a function of the form:

$$P_{2nd}(\alpha,\beta,\gamma)=\left(\frac{1}{2}-\frac{\beta}{2}-\gamma\right)^2+(1-\gamma)^2$$

Were the first term come from comparing the second derivative terms and the second term comes from comparing mixed derivative terms.

S2. Minimum of noise performance function

We wish to minimize

$$P_{N,WTnoisy}(\alpha,\beta,\gamma)=\sigma\sqrt{\alpha^2+3\beta^2+3\gamma^2}$$

Under the condition

$$\alpha+3\beta+3\gamma=1$$

Substitute $\alpha=1-3\beta-3\gamma$ to get:

$$P_{N,WTnoisy}(\alpha,\beta,\gamma)=\sigma\sqrt{(1-3\beta-3\gamma)^2+3\beta^2+3\gamma^2}$$

To minimize this expression, we can ignore the square root and take derivatives:

$$g(\beta,\gamma)=(1-3\beta-3\gamma)^2+3\beta^2+3\gamma^2$$

$$\frac{\partial g}{\partial \beta}=-6(1-3\beta-3\gamma)+6\beta$$

$$\frac{\partial g}{\partial \gamma}=-6(1-3\beta-3\gamma)+6\gamma$$

Equating both derivatives to zero we find: $\beta=\gamma=\frac{1}{7}$.

For

$$P_{N,WT=1}(\alpha,\beta,\gamma) = \sigma\sqrt{3\beta^2 + 3\gamma^2}$$

$\beta = \gamma = 0$ give the minimum.

S3. The Pareto front of noise and first order precision

We compute the Pareto front as the set of points for which the gradients of the performance functions are proportional (but in opposite directions), in the case of 1st order approximation performance the gradient is:

$$P_{1st}(\alpha,\beta,\gamma) = |1 - \beta - 2\gamma|$$

$$\nabla P_{1st}(\beta,\gamma) = \pm(1,2)$$

The noise function for noiseless wildtype is defined as:

$$P_{N,WT=1}(\beta,\gamma) = P_{N,DC}(\alpha,\beta,\gamma) = \sigma\sqrt{3\beta^2 + 3\gamma^2}$$

For simplicity we replace it by a function with the same contours:

$$P_{N,WT=1}(\beta,\gamma) = 3\beta^2 + 3\gamma^2$$

The noise gradient will therefore be in the case of drug combination:

$$\nabla P_{N,WT=1}(\beta,\gamma) = (6\beta, 6\gamma)$$

The Pareto front is then be defined by the equation (taking the gradients to be proportional):

$$\gamma = 2\beta$$

For the noisy wildtype case we have:

$$P_{N,WTnoisy}(\beta,\gamma) = P_{N,WTnoisy}(\alpha,\beta,\gamma) = \sigma\sqrt{(1 - 3\beta - 3\gamma)^2 + 3\beta^2 + 3\gamma^2}$$

Or in simpler form:

$$P_{N,WTnoisy}(\beta,\gamma) = (1 - 3\beta - 3\gamma)^2 + 3\beta^2 + 3\gamma^2$$

The gradient will be:

$$\nabla P_{N,WTnoisy}(\beta,\gamma) = (-6(1 - 3\beta - 3\gamma) + 6\beta, -6(1 - 3\beta - 3\gamma) + 6\gamma)$$

We get the equation:

$$-6(1 - 3\beta - 3\gamma) + 6\gamma = 2(-6(1 - 3\beta - 3\gamma) + 6\beta)$$

Simplify to get:

$$5\beta + 2\gamma = 1$$

S4. The Pareto front of noise and second order precision

The Pareto front is defined as the set of points which can't be improved at all performance functions at once. If we have two performance functions, it is simply the curve for which the gradients of the two performance functions are proportional to one another and point in opposite directions. This can be computed easily for the case of noise and second order performance functions.

For

$$P_{2nd}(\beta,\gamma) = P_{2nd}(\alpha,\beta,\gamma) = \left(\frac{1}{2} - \frac{\beta}{2} - \gamma\right)^2 + (1-\gamma)^2$$

And

$$P_{N,WT=1}(\beta,\gamma) = 3\beta^2 + 3\gamma^2$$

We can compute the gradients according to the parameters β, γ to get:

$$\nabla P_{2nd}(\beta,\gamma) = (-\frac{1}{2} + \frac{\beta}{2} + \gamma, -2(1-\gamma) + (-1+\beta+\gamma))$$

$$\nabla P_{N,WT=1}(\beta,\gamma) = (6\beta, 6\gamma)$$

Requiring the gradients to be proportional gives the following implicit representation of the Pareto front:

$$6\beta\big(-2(1-\gamma) + (-1+\beta+\gamma)\big) = 6\gamma(-\frac{1}{2} + \frac{\beta}{2} + \gamma)$$

Or:

$$-2\gamma^2 + 7\beta\gamma + 2\beta^2 + \gamma - 6\beta = 0$$

If we take instead the function for noisy wild-type:

$$P_{N,WTnoisy}(\beta,\gamma) = (1-3\beta-3\gamma)^2 + 3\beta^2 + 3\gamma^2$$

$$\nabla P_{N,WTnoisy}(\beta,\gamma) = (-6(1-3\beta-3\gamma) + 6\beta, -6(1-3\beta-3\gamma) + 6\gamma)$$

The equation for the Pareto front becomes:

$$(-6(1-3\beta-3\gamma) + 6\beta)\big(-2(1-\gamma) + (-1+\beta+2\gamma)\big) = (-6(1-3\beta-3\gamma) + 6\gamma)(-\frac{1}{2} + \frac{\beta}{2} + \gamma)$$

Or:

$$5\beta^2 + 28\beta\gamma - 22\beta + 16\gamma^2 - 20\gamma + 5 = 0$$

S5. Performance function for non-independent measurement noise

In the main text we analyzed the noise-robustness performance function in the case in which noise in the different measurements is independent. Here we consider the case of a general noise covariance matrix.

Log-linear formulae are linear combinations of the variables, therefore their variance can be computed using the covariance matrix:

$$P_{N,covariance}(\alpha,\beta,\gamma)^2 = Var\Big(\alpha L_\emptyset + \beta(L_1+L_2+L_3) + \gamma(L_{12}+L_{13}+L_{23})\Big) =$$

$$= \alpha^2 Var(L_\emptyset) + \beta^2 \sum Var(L_i) + \gamma^2 \sum Var(L_{ij}) + 2\alpha\beta \sum Cov(L_\emptyset, L_i) + 2\alpha\gamma \sum Cov(L_\emptyset, L_{ij}) + 2\beta\gamma \sum Cov(L_i, L_{jk})$$

We find that the performance function for this case is also quadratic in α, β, γ and its equi-performance contours are ellipsoids around the origin in the three dimensional space of α, β, γ. The analysis is therefore analogous to that described in the main text.

S6. Generalization to combinations of higher order

In the main text we claim that our approach helps to overcome the exponential explosion problem of number of combinations, and the main text demonstrates the case $n = 3$. We briefly discuss here the generalization to higher orders of interaction. We treated this problem from a mathematical perspective elsewhere (Tendler & Alon, 2018).

The aim is to estimate the effect of k perturbations using only the single and pair effects. One can define a family of log-linear formulae as:

$$L_{1\ldots k} = \alpha L_\emptyset + \beta \sum L_i + \gamma \sum L_{ij}$$

The noise performance function in this case is analogous to the triplet case:

$$P_{N,WTnoise}(\alpha,\beta,\gamma) = \sigma \sqrt{\alpha^2 + k\beta^2 + \frac{k(k-1)}{2}\gamma^2}$$

And in case II (noiseless $L_\emptyset$), depends only on the parameters β and γ:

$$P_{N,WT=1}(\alpha,\beta,\gamma) = \sigma \sqrt{k\beta^2 + \frac{k(k-1)}{2}\gamma^2}$$

The contours of this general performance functions are ellipsoids as in the triplet case.

The zeroth order precision condition is also defined analogously by the zeroth order Taylor approximation as:

$$1 = \alpha + k\beta + \frac{k(k-1)}{2}\gamma$$

It is a line in the 3-dimensional space of formulae, and its exact form depends on the order of combination we try to estimate.

The first order performance function is defined as the deviation of the first order Taylor approximation yielding (assuming zeroth order precision):

$$P_{1st}(\alpha,\beta,\gamma) = (1-\beta-(k-1)\gamma)^2$$

As in the triplet case the contours are lines, whose slope depends on k.

Finally, for the second-order precision we compute the deviation from the second order Taylor series. The generalized performance function is:

$$P_{2nd}(\alpha,\beta,\gamma) = k\left(\frac{1}{2}-\frac{\beta}{2}-\frac{k-1}{2}\gamma\right)^2 + \frac{k(k-1)}{2}(1-\gamma)^2$$

As in the triplet case, there is a single regression formula which is precise to the second order, and the performance contours are ellipsoids around it. The second-order-precise formula is:

$$Reg_k = \sum s_{ij} - (k-2)\sum s_i + \frac{k^2-3k+2}{2} s_\emptyset$$

S7. Synthetically generated datasets

To generate data synthetically we used randomly generated functions of third order of the following form:

$$f(x,y,z) = a_1 + a_2(x+y+z) + a_3(x^2+y^2+z^2) + a_4(x^3+y^3+z^3) + a_5 xyz + a_6(xy+xz+yz) + a_7(x^2y+xy^2+x^2z+xz^2+y^2z+yz^2)$$

Were a_i are chosen randomly from the uniform distribution on $[0,1]$. The function was evaluated at $x, y, z \in \{0,\epsilon\}$. So ϵ is a measure for the distance of evaluation of the function, which we called "interaction strength" in our graphs. To all evaluations of the function we added Gaussian noise of STD σ, which is the measurement of noise in the related plots.

For each value of ϵ and σ we took 10 different random functions, from each random function we generated 300 data points by choosing random values for x, y, z uniformly from $[0,1]$. For each dataset we computed the optimal formula, we obtain the optimal formula for the values of ϵ, σ by averaging the optimal formulae predicting the 10 functions.

We note that the distance of evaluation deserves the name "interaction strength". It is indeed related to a natural definition of interaction strength as $\frac{|s_{ij}s_\emptyset - s_i s_j|}{|s_i s_j|}$. Fig A shows the relation between the two quantities on a simulated dataset.

S8. The bias-variance tradeoff in the generalized mean class of formulae

In the main text we analyzed the log-linear class of formula. Here we analyze another class of formula, the class of *generalized means* defined as:

$$s_{123} = GM_p = \left[\frac{1}{3}(s_1 s_{23})^p + \frac{1}{3}(s_2 s_{13})^p + \frac{1}{3}(s_3 s_{12})^p\right]^{\frac{1}{p}}$$

Where p is a parameter. For p=-1 this is the harmonic mean of terms s_i s_{jk}, and for p=1 it's their arithmetic mean. Each of these terms is similar to a Bliss approximation taking a single s_i and the other pair s_{jk}, so the generalized mean is away to account for all three Bliss-like possibilities. At certain limits of p the generalized mean has the following forms:

$$GM_{-\infty} = \min\,(s_1 s_{23}, s_2 s_{13}, s_3 s_{12})$$

$$GM_0 = (s_1 s_{23} s_2 s_{13} s_3 s_{12})^{\frac{1}{3}}$$

$$GM_{\infty} = \max\,(s_1 s_{23}, s_2 s_{13}, s_3 s_{12})$$

All GM formulae are accurate to the zeroth order. Indeed, if we plug in $s_1 = s_2 = s_3 = s_{12} = s_{13} = s_{23} = 1$ we get $s_{123} = 1$.

Moreover, the generalized means are precise to the first order: assuming $s_{12} = s_1 s_2, s_{13} = s_1 s_3, s_{23} = s_2 s_3$ we get:

$$s_{123} = GM_p = \left[\frac{1}{3}(s_1 s_2 s_3)^p + \frac{1}{3}(s_2 s_1 s_3)^p + \frac{1}{3}(s_3 s_1 s_2)^p\right]^{\frac{1}{p}} = s_1 s_2 s_3$$

These formulae are also precise to the second order, this can be seen by computing the Taylor series of both sides of the equation (not shown).

The generalized mean formulae differ in their noise robustness. Noise grows with p which makes the *min* model ($GM_{-\infty}$) the most noise-robust (Fig B). As suggested by the noise robustness analysis, the min model provides the best predictions in this class for some datasets (Extra data on A549, H1299 (Zimmer, Tendler, Katzir, Mayo, & Alon, 2017) and yeast three-gene deletion interactions (Kuzmin et al., 2018), not shown).

Generalized means differ also by their prediction about the type of interactions, because of the *mean inequality*: if $p < q$ then $GM_p \leq GM_q$. Therefore, the min model will be favored for synergistic interactions, whereas the max model (GM_{∞}) will be preferred if most interactions in the dataset are antagonistic. In the present study, all non-expanded datasets (Table 1) showed a majority of antagonistic interactions ($s_i s_j < s_{ij}$). The only exception was (Beppler et al., 2016), which was constructed as synergistic.

This suggests a bias-variance tradeoff among the generalized mean formulae: the max model (GM_{∞}) has low bias since it predicts antagonism and most datasets are antagonistic, whereas the min ($GM_{-\infty}$)model has low variance since it is the most noise-robust.

We compared the generalized mean class to the log-linear class for the present datasets (Fig 2SB). The generalized mean class performed significantly better on only one of the datasets studied here, the yeast triple gene deletion dataset. The best GM model showed a RMSE of 0.09, compared with 0.11 for the best loglinear formula, and 0.13 for the Isserlis model used in the original study as a null model.

References

Beppler, C., Tekin, E., Mao, Z., White, C., McDiarmid, C., Vargas, E., … Yeh, P. J. (2016). Uncovering emergent interactions in three-way combinations of stressors. *Journal of the Royal Society, Interface*, *13*(125), 20160800. https://doi.org/10.1098/rsif.2016.0800

Franke, J., Klözer, A., de Visser, J. A. G. M., & Krug, J. (2011). Evolutionary Accessibility of Mutational Pathways. *PLoS Computational Biology*, *7*(8), e1002134. https://doi.org/10.1371/journal.pcbi.1002134

Kuzmin, E., VanderSluis, B., Wang, W., Tan, G., Deshpande, R., Chen, Y., … Myers, C. L. (2018). Systematic analysis of complex genetic interactions. *Science (New York, N.Y.)*, *360*(6386), eaao1729. https://doi.org/10.1126/science.aao1729

Lozovsky, E. R., Chookajorn, T., Brown, K. M., Imwong, M., Shaw, P. J., Kamchonwongpaisan, S., … Hartl, D. L. (2009). Stepwise acquisition of pyrimethamine resistance in the malaria parasite. *Proceedings of the National Academy of Sciences of the United States of America*, *106*(29), 12025–12030. https://doi.org/10.1073/pnas.0905922106

Tendler, A., & Alon, U. (2018). Approximating Functions on Boxes. Retrieved from http://arxiv.org/abs/1804.02045

Weinreich, D. M., Delaney, N. F., Depristo, M. A., & Hartl, D. L. (2006). Darwinian Evolution Can Follow Only Very Few Mutational Paths to Fitter Proteins. *Science*, *312*(5770), 111–114. https://doi.org/10.1126/science.1123539

Wood, K., Nishida, S., Sontag, E. D., & Cluzel, P. (2012). Mechanism-independent method for predicting response to multidrug combinations in bacteria. *Proceedings of the National Academy of Sciences of the United States of America*, *109*(30), 12254–12259. https://doi.org/10.1073/pnas.1201281109

Zimmer, A., Katzir, I., Dekel, E., Mayo, A. E., & Alon, U. (2016). Prediction of multidimensional drug dose responses based on measurements of drug pairs. *Proceedings of the National Academy of Sciences*, (32), 201606301. https://doi.org/10.1073/pnas.1606301113

Zimmer, A., Tendler, A., Katzir, I., Mayo, A., & Alon, U. (2017). Prediction of drug cocktail effects when the number of measurements is limited. *PLOS Biology*, *15*(10), e2002518. https://doi.org/10.1371/journal.pbio.2002518

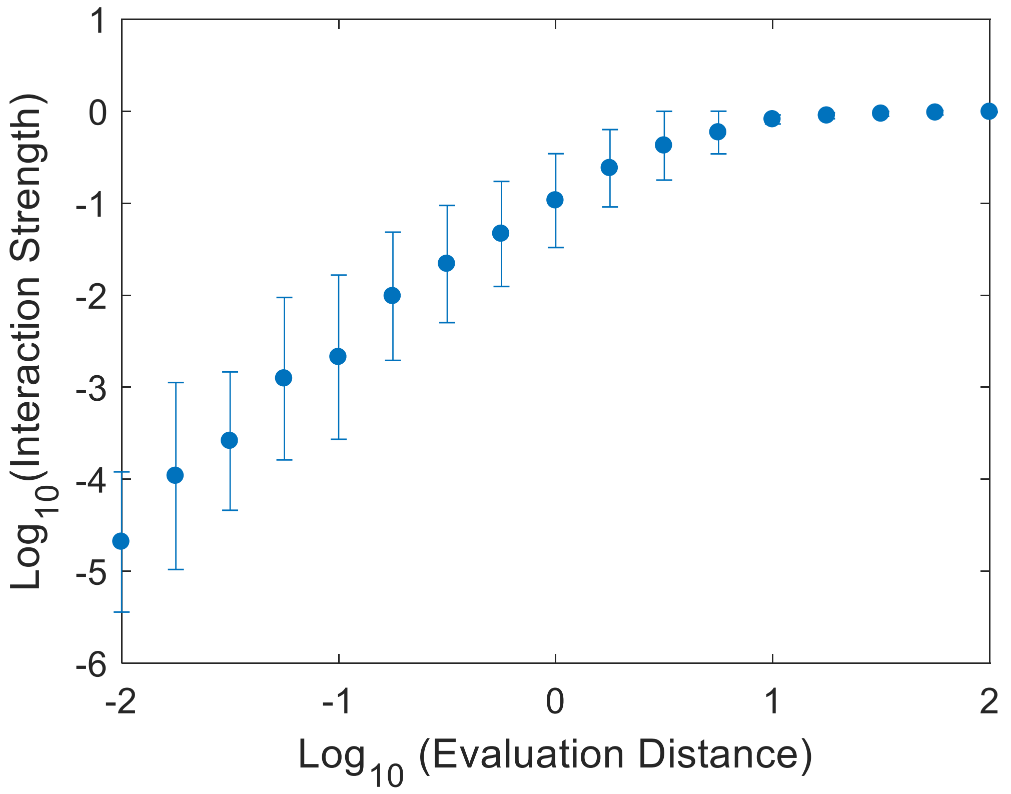

Fig A. interaction strength is related to evaluation distance. For random simulated functions with difference evaluation distance we computed the resulted interaction strength as computed from the dataset. Each point is an average of 100 simulations, errorbars are standard deviation of the 100 results.

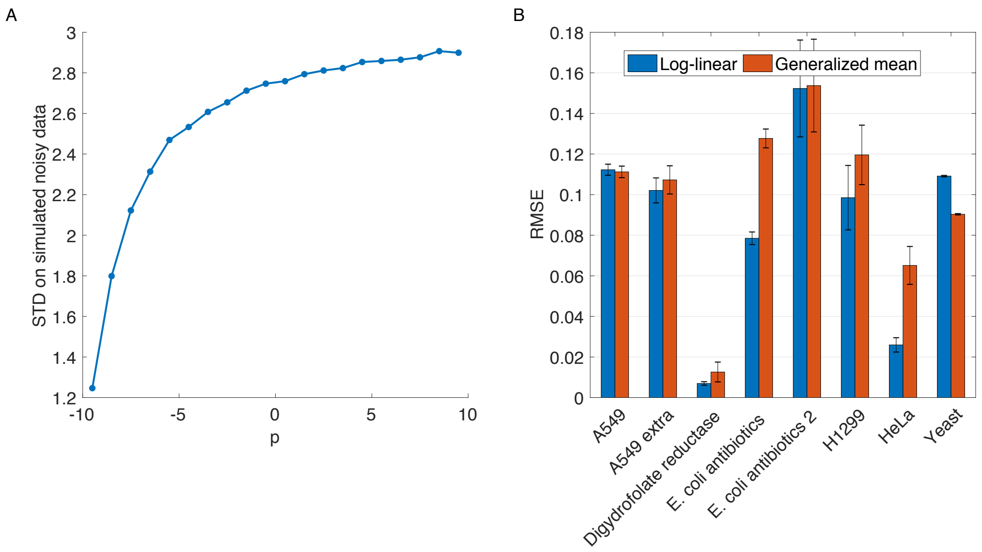

Fig B. Analysis of the generalized mean class of formulae. (A) Noise increases with the parameter p. The horizontal axis is the parameter p of the generalized mean model and the vertical axis is the standard deviation of the predictions of the model. Each point is based on 100000 lognormally distributed s_i, s_{ij} (log of si and sij has mean zero and standard deviation of 1). The model becomes more sensitive to noise as p grows larger. **(B)** Comparison of the performance of log-linear and generalize mean classes. For each dataset we computed the optimal formula in the log-linear and generalize mean classes, we compare the performance of the optimal formulae in terms of root mean square error. Error-bars result from bootstrapping each dataset 100 times.
